# Statement of the Eruptive Disease as It Prevailed in Hornellsville, Steuben Co., N.Y.

**Published:** 1854-12

**Authors:** W. H. Renale


### BUFFALO MEDICAL JOURNAL

AND

### MONTHLY REVIEW.

VOL. 10. DECEMBER, 1854. NO. 7.

### ORIGINAL COMMUNICATIONS.

ART. I.—*Statement of the Eruptive Disease as it prevailed in Hornellsville, Steuben Co., N. Y.* By W. H. RENALE, M. D.

DOCT. HUNT:

*Dear Sir,*—As a subscriber to the Buffalo Medical Journal, permit me to request you to give the following statement of the eruptive disease as it prevailed in Hornellsville, Steuben Co., N. Y., for the last seven or eight months subsequent to January. I should not have troubled you with this communication but for the fact that I see in your Journal of September last, an article to this effect: "Reply to Dr. Renale, in relation to an eruptive disease at Hornellsville. By Frank H. Hamilton."

I am constrained, by three considerations, to notice Professor Hamilton's reply to my statement made in a weekly paper, (Dansville Herald,) of June last, in relation to an eruptive disease then prevailing in Hornellsville:

*First.* On account of a paper that he read before the Buffalo Medical Society on that subject.

*Second.* On account of an erroneous statement that he made, by saying (in the Medical Journal) that the two cases which he saw and examined at a Mr. Wilson's house, in Hornellsville, and called varicella, I had seen and named them varioloid or variola.

*Third.* By saying that Dr. Wisewell regarded his opinion on the Wilson cases, as *decisive* of the *whole* question, whether variola was present in Hornellsville or not.

We will see how far the above statements are correct. Dr. Hamilton, after giving the history of two cases of eruptive diseases which he saw at the house of a Mr. Wilson, in Hornellsville, and describing them minutely, winds up by pronouncing them to be varicella; he says Dr. Renale called them varioloid or variola. As regards Mr. Wilson I have never seen the man to know him, nor any of his family, nor any of the inmates of his house, as named by Prof. Hamilton; he and they are *all* strangers to me, except by notoriety. I never *saw* nor *named* a disease under which any of them ever labored. Had Dr. Hamilton read my article published in the Dansville Herald, of June last, in reply to my friend Dr. Robinson, as carefully as he thought he did, he would not have committed such an error, (for in that article I denied having seen Mr. Wilson's children; my language is, "this cannot be so;") and have placed me in the awkward position that he has before the medical world and a discerning public, by saying that I called chicken-pox varioloid or variola, cases which *never* came under my observation. How far Prof. Hamilton and Dr. Wisewell were correct as regarded the diagnosis of the Hornellsville epidemic, we will leave your readers to judge after canvassing the subject.

On the 12th day of last April, I received a letter from Hornellsville, signed by seven of her most respectable citizens, bearing a request for me to visit their place and give my opinion respecting the nature of an eruptive disease then prevailing in their village, about which physicians were divided, some calling it chicken-pox, whilst others called it small-pox, saying that my opinion would be satisfactory on the question at issue. On the next day, April 13, I went to Hornellsville, examined three cases most *carefully* and *accurately*. The first case, little Miss Barnard, with distinct small-pox. 2. Miss Sarah Burley, aged, I think, 23 years, with confluent small-pox. 3. A child of Esq. Height's, about 2 years of age, with varioloid.

We will return to this part of our subject again. Dr. Hamilton's description of S. L. Wilson's child begins on the twelfth day of the month, the day before my visit to Hornellsville; read the two cases as he describes them, and it will be readily perceived that his second case, the little girl, accords, to say the least, as well with the varioloid disease as it does with that of chicken-pox; the eruption appeared first on the neck, face, and head, and then on the body, &c. This is not the *usual* place for the chicken-pox eruption first to show itself. In the boy, the place where the eruption commenced might be claimed by either party to be varicella or varioloid, as it was first observed on the front of his neck, back, forehead, &c. Now the description of these two cases, as related by Prof. Hamilton, notwithstanding their mildness, are not incompatible with the varioloid disease, either in their mildness or eruptive duration, particularly when it is remembered that the Hornellsville epidemic was of a variolous nature, as I expect fully to prove. I was in Hornellsville on the 13th day of April; the eruption commenced on Mr. Wilson's children the day previous, according to Dr. Hamilton's statement, so that it will be perceived that even if I had seen Mr. Wilson's children, (which I did not,) it would have been very difficult to have decided upon the nature of the disease. Good says, page 631: The chicken-pox, according to Drs. Mohl, Abercrombie, and Mr. Bryce, *never* assumes its vesicular character before the second day; on the first they never observed it. Wood, page 377, third edition, speaking of varioloid, says: Sometimes it never advanced beyond the state of mere *papula or pimple.* On the same page he says: Notwithstanding the generally protective or modifying influence of previous vaccination or variola, it must be confessed that genuine small-pox, perfect in all its stages, has sometimes followed those diseases; while on the other hand, modified forms of the complaint have been noticed in persons who had never been affected with either of them. Again, page 381: Between certain cases of varioloid, and varicella, or chicken-pox, there is *absolutely no observable difference;* but in general, the two complaints may be readily distinguished by the much shorter duration of the eruptive fever of the latter, and by the absence of umbilicated vescicles or pustules. Dunglison, page 566, second vol., second edition, speaking of varioloid, says: Doubtless the varying character of the eruption and the shortened duration of the disease, first gave the impression that it was varicella, or chicken pox, which as elsewhere shown, varies very materially in the phenomena it presents. Dr. Lee, of the Buffalo Medical School, says, in the Buffalo Medical Journal for October, 1853, page 291, speaking of the Gorham epidemic: An anomaly was observed in several instances in the disease, which served still more to render the diagnosis difficult, and that was, individuals who had not been vaccinated, *had the disease in a very mild form*, indeed no one could distinguish them from varicella. This, however, has been noticed in other epidemics. Page 293, he says: It would be wrong, however, to infer that the controling power of the vaccine disease was not exhibited in this epidemic, for as a general rule the eruption was of a more variceloid form, and the constitutional symptoms much milder, in cases where vaccination had previously been performed, though there were some *striking remarkable exceptions to this.* Read also his seventeen points, at page 294. Dr. Lee's whole article is full of interest on this subject; *I look upon it as parallel* with the Hornellsville epidemic.

From the above standard authors, and the observation of many critical observers, it will be seen how difficult it is sometimes to say positively, in isolated cases, (Prof. Hamilton saw but two,) whether the disease is varioloid or varicella. Esq. Height's child, I am very sanguine, had the varioloid; I saw it on the third day of the eruption; premonitory symptoms had not been very severe, vesicles filled with a serous fluid, and ten or twelve of them *distinctly* umbilicated on the face and breast. Drs. Truesdell and Ward observed them, as well as the father and mother. Mr. Wilson, (I am told,) lived in the house with Esq. Height, but in a different apartment. I am *strongly inclined* to the belief that both families had the same disease, especially as the nature of the epidemic was of a variolous nature, modified by vaccination, atmospheric and other constitutional causes. Height's child had been vaccinated. I saw and examined Miss Sarah Burley on the eleventh day of the eruption, and the fourteenth from the commencement of the disease, and one day before she died. I found her covered, from head to foot, with a pustular disease such as medical writers describe as being small-pox, (a disease that I have frequently seen and treated for the last thirty-three years.) The umbilicated depression was well marked in all the isolated pustules so peculiar to small-pox; but of course where they coalesced, as on the face, front of the neck, &c., the central depression was not so perfect, yet was plain to be seen even where the eruption was confluent. Her lips, both on the outside and inside of her mouth, tongue, fauces, &c., were studded with small-pox pustules; eyelids, face, and head badly swollen; delirium; pulse 120, and feeble; patient evidently typhoid and sinking; and that peculiarly disagreeable odor that exhales from the throat and surface of the body of a patient laboring under malignant confluent small-pox, was most distinctly present. It is a strong diagnostic symptom of the disease, one that cannot be mistaken for chicken-pox. Her throat was very sore and attended with great hoarseness; deglutition almost impossible; salivation had been profuse, but now declining a little. Upon inquiry they informed me that the premonitory symptoms had been very severe, especially as regarded the gastric irritation and lumbar pains. I was further told that the eruption first appeared upon the face, as forehead, cheeks, lips, &c.; next on the neck and arms, then on the trunk of the body, and so on downward. I found this patient's face, hands, and front of her neck, *covered with collodion*, a strange remedy for chicken-pox, but good for the disease for which it was applied.

The case of the little Miss Barnard, (seven years of age,) was equally as legitimate a case of small pox as the above described one, only the pustules were distinct—she carries the unmistakable marks of the disease in her face. She commenced desiccating on the tenth or eleventh day of the eruption. Neither of those patients had ever been vaccinated.

At my request, Dr. Alley, of Almond, Alleghany Co., N. Y., writes me thus, Sept. 25, 1854: On the 26th of last May, I saw two of Mr. Coburn's family, the first that were taken sick with the disease, (small-pox.) It was on the tenth day of the eruption, and the thirteenth day of their sickness. Both have previously had the chicken-pox. Neither had ever been vaccinated. The girl, aged 15 years, I found covered with a pustular eruption, from the top of the head to the bottom of the feet, mostly confluent. The pustules on the face were covered with a brownish crust; matter was oozing from the face and scalp; the odor was almost intolerable, notwithstanding the windo s were open and floor strewed with chloride of lime; the tumefaction of the face and extremities was considerable. The distinct pustules on the feet had a central depression; I punctured several, a whitish-yellow substance exuded, still the pustules remained above the surrounding skin. Febrile symptoms severe; pulse 112, and active; skin dry; eyes red; tongue brown; inside of the mouth studded with pustules; pain and soreness in the throat excessive; moderate cough; drules considerably; could not speak aloud; mind lucid. The boy appeared very much like the girl, except more stupid. Pulse 120; eruption semi-confluent; eyelids closed and badly swollen; the umbilicated depressions were well developed in the distinct pustules; mind confused and considerable subsultus; desiccation had not commenced in either case.

Dr. Truesdell, of Hornellsville, writes me thus, Sept. 27, 1854: On the 14th day of last May, I was sent for to visit Charlotte Coburn, aged fifteen years; I found her laboring under all the premonitory symptoms of the eruptive disease then prevailing in our place. The pain in the head, stomach, and back, was excessively severe; third day small red spots appeared on the face, neck, and hands; tongue red; pulse 125; throat sore. Sixth day the distinct pustules are umbilicated, but on the face the eruption is almost entirely confluent. Eighth day, face and neck much swollen; deglutition almost impossible; pustules are filling on the extremities, and depressed in the center. Ninth day, the eyes are closed; very hoarse and restless; delirious. Tenth day, swelling of lower extremities; drowsiness; complains much of smarting and burning heat of the whole body; pulse 98; inside of the mouth studded with pustules. Thirteenth day, the whole body covered with pustules from head to feet; stench almost intolerable; chloride of lime is strewed all about the house; tumefaction of face diminishing, and extremities becoming swollen. Some of the pustules of the face have purulent matter exuding from them; some I opened and pressed out their contents, but still the hard elevated base remained; patient rational; pulse 115. Seventeenth day, desquamation is proceeding slowly on the face and breast; pulse 100; some appetite. On the extremities the pustules are flattening down, and the scabs are falling off the face rapidly. Twenty-first day, desiccation well advanced and patient doing well. Twenty-eighth day, patient well scarred and pitted. A boy, five years old, was taken sick the next day after the girl was attacked. He recovered more slowly and is badly pitted. There was no great difference in the progress of these two cases, except a little more severity of symptoms in the boy.

Between the 29th of May and June 2d, four more were attacked with variola, and the father and mother with the varioloid, making six cases of variola and two of varioloid. Of those last taken sick, one, a ricketty subject, was more severely attacked than any of the others had been; the eruption being entirely confluent over the whole body. He died on the tenth day of the eruption. The stench was so intolerable for two days before he died, that no one could be near him for any length of time. The countenance became almost black. The children were all unprotected by vaccination. The father and mother had been vaccinated about fifteen years before. The children had *all* had the chicken-pox. There was one girl, (Sarah,) that had the evidence of a good vaccination years before; she was attacked with what Drs. Hovey, Ackley, and myself, considered a variolous fever; no eruption appeared.

Dr. Ward, of Hornellsville, writes me as follows: Sept. 29, 1854. The following are some of the prominent symptoms of Margaret Pindall, aged 12 years; first visit March 12th. The Dr. goes on and describes the premonitory symptoms of the eruptive disease, very minutely, from time to time, (all bad enough,) until he comes to the third day of the disease, when he says: "bright red spots appeared upon the face, with less fever. Fourth day, the eruption had extended to the neck, body, and upper extremities. Fifth day, the eruption covers nearly the whole body, and on the face the vesicles contain a little clear lymph. Sixth day, the vesicles increase in size, and on the face commence flattening on the summit, and some of them a little depressed in the center. Seventh day, pustules more umbilicated on the trunk and extremities, but become fuller on the face. Ninth day, contents of the pustules becoming opaque on the face, and more convex. Eleventh day, pustules begin to mature on the whole surface of the body; the face somewhat swollen; pock distinct on the arms but so large and near together that it is difficult to feel the pulse; soreness of throat, with a peculiarly disagreeable smell. Twelfth day, some little fever; the pustules begin to scab and turn brown on the face. Fourteenth day, the scabs on the face are drying down; feet and hands swollen; patient comfortable. Sixteenth day, pock begin to dry down on the extremities; tongue clean, and patient begins to have some appetite. Eighteenth day, some of the scabs are falling off from the face; appetite increases, but the patient continues to emit a very unpleasant and disagreeable odor. The patient continued to improve, and in about ten days more was well. This patient had never been vaccinated. She had the chicken-pox about three years ago. Margaret had been at the Western Hotel, (Cobb's,) about the first of March; this is the house that had the *first case* of eruptive disease in dispute; some were there sick in the house, and from them she took the disease. Margaret had two younger sisters unprotected by vaccination. They were vaccinated, but from some defect in the vaccine virus not working perfectly, in about two weeks they came down with the same disease, but it exhibited a light character, so much so that they required but little treatment. The father, mother, and two brothers had the varioloid disease several years before; they all escaped unharmed; but a little boy and the Barnard girl, were exposed at Prindall's, and both had the disease severely. The Barnard girl is the one you saw on the day you visited Miss Sarah Burley. I could give a description of some twenty-five or thirty cases more."

I received a letter from Dr. Hess, of Haskensville, Steuben Co., N. Y., dated October 4, 1854. He gives a very minute and excellent description of a family by the name of Travis, who reside about three and a-half miles east of Hornellsville, from which place the disease was taken to them. I am sorry that want of room will not enable me to give the Dr.'s language entire, for it is very interesting. We will pass over the premonitory symptoms which the Dr. describes as being very severe. He says: the pustules were irregular in shape, and became confluent over the entire surface of his face, neck, a portion of his chest and extremities; on the bowels they were quite distinct, and they were generally both umbilicated and cellular. As the disease advanced the pustules changed from a milky to a more yellowish color; but they did not fill well; they commenced drying on the tenth day after their first appearance; on the eleventh they were considerably dried upon the face, as you saw when visiting him with me; and on the eve of the twelfth day he died.

From the ninth until the day of his death, the stench was disgustingly offensive; after his death, the offensive effluvia could be distinctly detected to the road, which is about three or four rods from the house. Two other children of the family commenced complaining about this time; they were unprotected by vaccination; the operation was repeatedly performed, but the virus proved not to be good, and the consequence was they had the small-pox the natural way. The eruption first made its appearance upon the forehead, face, mouth, &c.; next upon the neck, arms, chest, and abdomen; and lastly upon the lower extremities. When first seen they were small red spots; the next day they were a little elevated, and some inflamed about the base; the third day a portion of them showed a central, and on the next day the characteristic diagnosis was present in nearly all of them. At first they were filled with a limpid watery fluid; and about the fourth day it changed to a milky or white color; and on the ninth day they were filled with a yellowish purulent matter. The face was very much swollen, and her eyes entirely closed for a number of days; the swelling began to subside in her face about the tenth day, and her body, hands, and feet, were more swollen after that time than before. As the swelling subsided from the face, the pustules began to shrink, and when they dropped off they were a dry, hard, brown scab. The other unprotected child ran a similar course, but was not quite as severe a case. This family had had the chicken-pox some years ago. There were three or four more in the family that had some pustules, but they dried much sooner than those who were unprotected; and there was one that had very strong symptoms, high fever, pain in the back, &c., but no eruption appeared; this accords with our best writers on the subject.

Dr. Ackley, of Hornellsville, under date of October 2, 1854, writes me thus: I visited —— Stratten, in July last, in company with Dr. A. B. Case, the day before he died. He was an awful object to look upon; face enormously swollen; eyelids closed; mouth, tongue, and lips studded with small-pox. The disease was confluent on the face, neck, and body; commenced desiccating. The peculiarly disagreeable odor that exhaled from his body, so diagnostic of the disease, was almost overpowering. He *was* decidedly the most disgusting and loathsome mass of human putrefaction, of which the mind can well conceive. He was Dr. Wisewell's patient. I visited Miss Sarah Burley, on several occasions during her sickness. Her disease was confluent small-pox, of as bad a character as ever destroyed a human being, in my judgment; face, eyelids, and whole body, badly swollen; peculiar odor distinctly present; mouth and throat thickly studded with pustules; great hoarseness and difficulty of deglutition. I also visited the little Miss Barnard on several occasions; disease distinct small-pox; she is pretty well pitted, distinctly so.

I think I have proven, by the best of testimony, that small-pox prevailed in Hornellsville last winter, spring, and summer. I am now going to offer proof equally strong, to show that the eruptive disease, as it lately prevailed in Hornellsville, and some of the cases no worse than the two which Dr. Hamilton examined at Mr. Wilson's, and called varicella, were of a varioloid nature, and would *produce genuine small-pox* in the unprotected; *varioloid* in the partially protected, and *no disturbance* of the constitution, where the vaccine impression was good in the system. To this end permit me to transcribe from a letter that I received from Dr. J. Whitney, of Hunt's Hollow, Livingston Co., N. Y., dated June 29, 1854. After some introductory remarks the Dr. says: "A Mr. Lockwood and lady, came up from Hornellsville to visit their friends at this place; the lady had the disease supposed to be chicken-pox, and thought there would be no danger, for the doctors told her so. After about a fortnight, a child of Mr. Nelson Lockwood, came down with the disease. I attended the case all through, and observed its cause, particularly to satisfy myself as to its nature. The child had an intense fever of three days' duration, the last of which was attended with convulsions. The fever ceased after the accession of the eruption, which was first papular, then vesicular, and lastly pustular. It was distinctly umbilicated. A secondary fever came on about the eighth day; it was also severe, from the fact the eruption was very profuse; being confluent upon the face, neck, breast, and upper extremities; we came to the conclusion that the disease was variola, that it was a *typical* case, corresponding *precisely* with the description of variola in the books. In fact every symptom belonging to small-pox was present, while none were present of chicken-pox.

"It was communicated to two of the family, who having been previously vaccinated, had a varioloid. Two other children whom I vaccinated recently, escaped altogether; at least they have not as yet any symptoms which they should, if they were going to have the disease, proving that it does not attack those who have been vaccinated, as was alledged in Hornellsville. We took every precaution to prevent its spreading, and up to this time there have been no more cases. Two other physicians have seen the cases and are of the same opinion with me—Drs. DeCamp and Kneeland."

Here is a plain case not to be mistaken. Mrs. Lockwood had been laboring under mild *varioloid*—called *chicken-pox* by her medical attendant—a great error in diagnosis, for chicken-pox cannot produce small-pox, as the two diseases are specifically different. The effluvia which emanated from that woman, produced in the unprotected, confluent small-pox; in the previously vaccinated, varioloid; and the unprotected were fully saved by immediate vaccination. How plain the case is! what unanswerable truth it sets forth!

There can, I think, be no doubt but this disease originated in Mr. Cobb's house, (the Western Hotel, in Hornellsville,) sometime in January last. I saw and conversed with Mr. Cobb on this subject, in Dansville, about the 25th of last June. Upon inquiry he told me, in the presence of two other medical gentlemen, that the eruption and pustules first appeared on his temples, forehead, and face, then on his body, &c., (not the place where the chicken-pox eruption first appears.) His description was clear and lucid; no leading questions were put to him. He said, after the eruption first came out he was soon better, and able to walk about. The marks could then be seen dimly, but distinctly, on his face. The disease was evidently varioloid. Mr. Cobb had been vaccinated many years before. His family and inmates had the disease; they took it, I suppose, from a Mr. Hawley, who had just returned from New York, and had the disease. I have no doubt, from this case, (Hawley's,) the disease originated, which has spread and been kept alive by epidemic and other influences, until it was eradicated from the place about the first of September last, through the instrumentality of a well organized and efficient Board of Health.

I have no doubt but varicella prevailed in Hornellsville and neighborhood last fall, and probably run somewhat into the cold weather; but that it continued to remain there during the winter, spring, and summer months, and run a parallel race with the variolous disease, I do not believe for one moment. When we remember what a *variety* of eruptive forms the varioloid disease assumes, according to the amount of protecting vaccine power that has left the system, from mere papula up to almost natural small-pox, we must not be astonished at the great variety of faces which the disease puts on.

This eruptive disease has spread "far and wide;" to the west as far as Arkansas and Wisconsin; and wherever it was carried by contagion, (received in Hornellsville,) east, west, or any other direction, the physicians have called it small-pox. I can count, myself, thirty-two medical men who have seen and examined this disease, (about which so much has been said and written, and which have produced so much commotion and trouble in its neighbornood, respecting its true nature,) and called it small-pox. My friend Dr. Alley, counts over forty.

WM. H. REYNALE.

DANSVILLE, Oct. 2, 1854.

DR. REYNALE:

*Sir*,—I will cheerfully comply with your request and briefly give the history of a few cases of the disease to which you refer:

On the second day of June last, I was in the village of Hornellsville, on professional business, and at the request of some medical gentlemen of that place, I visited quite a number of persons who were sick with the then prevailing disease of that village.

CASE I.—In company with Dr. Wisewell, we repaired to the house of a Mr. Bulkley; saw his child, who was about two years of age, sick with the eruptive disease. The history of the case as I have it from the doctor and mother of the child, was, that slight fever, restlessness, sickness of the stomach, attended the child two or three days before the eruption commenced, at which time its first appearance was on the face and neck, and then spread, covering the whole body and extremities. The pustules commenced by red points, and continued to increase in size up to about the sixth day, at which time I saw it, and they were then three to four lines in diameter. About the face the pustules ran into each other a little, but were distinct on the other parts of the body; they were elevated above the surrounding skin with a slight areola. Most of the pustules were oval, with but little depression, yet some presented the true umbilicated form, with a distinct depression in the center. The "*cuticle* appeared distended with a *semi-transparent fluid*" of nearly a straw color, and occasionally one had a small brown scale in the center. There was but little constitutional disturbance. I have it in my notes of the case, that the child had never been vaccinated.

I gave it as my opinion to Dr. Wisewell, that this was a case of distinct variola.

CASE II.—Mr. Minnick was a German, 28 years of age, of previous good constitution, (for I had known him in our village,) whom I saw in company with Dr. Ackley.

It was on the sixteenth day of the eruption that I saw this man. The history of the case as we had it from the nurse, was: that the premonitory fever was very severe, lasting for three days before the eruption, accompanied with sickness, vomiting, pain in the back, headache, thirst, &c. The eruption commenced about the face and forehead, extending to the neck, arms, and finally covering the whole body. His face at the time I saw him, was covered with a brown crust or scab, as desiccation was completed on the face, neck, and arms. The inferior extremities were very much swollen, and the pustules assumed very much the spherical form; many of them on the extremities, were one-half inch in diameter, and distinct; while those on the face and neck were confluent. This patient was situated in a well-ventilated room, yet the odor was almost beyond endurance, and of that peculiar character that is described by some as a diagnostic symptom of variola. The teeth were sordid; the tongue dry; deglutition very difficult; a thick viscid saliva; pulse 140; and for most of the time he had been delirious. Never had been vaccinated. This man died six days after my visit, on the twenty-second day of eruption.

It appears to me that all well-informed physicians would not hesitate in pronouncing this a case of *confluent* small-pox.

CASE III.—Was the daughter of Esq. Hale, a young lady. The history of this case I have from a physician who was on a visit at Mr. Hale's, a reside t of Maia.

This young lady had been vaccinated. The premonitory fever was not severe, though it continued for three days before the eruption. Its first appearance was on the face, and diffused itself over the whole body.

The eruption appeared first in small papulæ, and many dried off without becoming either vesicular or pustular. It was on the thirteenth day of the eruption that I saw this case, at which time most of the scabs had fallen off, leaving slight depression, or pits, with a peculiar redness of the skin. From the history and what I saw, I have no doubt that it was modified small-pox.

The Coburn family which Dr. Truesdale attended, (and he will be able to give the symptoms in detail,) is perhaps the most interesting, and will throw more light upon the question of diagnosis than any family who suffered with that disease.

On the day of my visit there were seven persons sick with the epidemic of that village, in this family. The two first who were taken were convalescing rapidly as desiccation was completed, it being on the seventeenth day of eruption. Five other members of the family were taken sick in about two weeks after the two first, and the difference in the appearance of the disease was no more than would be observed in measles or other cutaneous diseases of different persons who were exposed by the same contagion.

In the two younger, most of the pustules were nearly filled with a limpid fluid, while some about the face were changing to a purulent character.

There was considerable swelling of the face, soreness of mouth, and difficult deglutition.

A young man, about 18 years of age, the eruption had not advanced as far; though in this case the eruption presented small elevations, with inflamed basis, and but few contained any "lymph," and none purulent matter. The father and mother, who had been vaccinated, had but few pustules, which presented the usual appearance of those in modified small-pox. The last, a young lady, who had evidences of vaccination, was feverish, pain in head, back, and extremities, which I regarded to be a *variolous fever*.

Such is a brief statement of this family, and could a detailed statement of all the symptoms be presented to the medical world, I have no doubt but the most incredulous upon the "name" of the epidemic which prevailed in Hornellsville, would be impelled to acknowledge that *variola*, in some of its forms, was the *true* and *only* prevailing disease of that village. It will be noticed that the period of incubation from the first that were sick, was the usual time that all writers on variola fix as being the distinct period. It will also be observed, that vaccination modified the disease in all that had undergone its process, and that the others went through the usual stages: fever; papulæ; formation of lymph; then purulent matter; process of desiccation; which all who are familiar with the disease do universally observe.

Lest I weary your patience by my lengthy letter, I will close, though the subject is full of interest, and but a small part been said. If anything has been communicated to you that will answer the purpose for which you are seeking, you are at liberty to use as your judgment directs.

Very truly yours,

B. L. HOVEY.

